# Observations on Utero-Gestation, with Suggestions How to Arrive at More Correct Data in Respect to the Time Occupied

**Published:** 1848-09

**Authors:** C. Clay

**Affiliations:** Manchester, Editor of the British Record of Obstetric Medicine, etc.


***Observations on Utero-Gestation, with suggestions how to arrive at more correct data in respect to the time occupied.*** By C. Clay, M.D., Manchester, Editor of the British Record of Obstetric Medicine, etc—I have been, for some years, pursuing a series of enquiries into the probable length of the term of utero-gestation, in order, if possible, to arrive at some definite conclusion respecting that much-debated question, and, if possible, ascertain the established laws by which its duration is governed. Hitherto this subject has been exposed to the most conflicting opinions, no two authorities having yet agreed; nor is it likely that anything approaching unanimity can prevail, until some clue is discovered of the existing laws of nature by which the question at issue is regulated, and which has, as yet, escaped the notice of physiologists. The difficulty of obtaining direct evidence respecting correct terms of utero-gestation (on account of the peculiar nature of the enquiry in respect to the human species) will always militate against the accumulation of a large amount of proofs: still there are such on record which cannot for a moment be doubted; and when those, with other general observations, are supported by facts resulting from enquiries and experiments on the domesticated animals lower in the scale of creation, I conceive that we can promulgate a law approaching much nearer the standard of truth than any yet admitted. I have in my possession a considerable amount of evidence, extending over many years of my past life, which fully bears out my opinions, although I cannot at present enter into a minute detail of all the cases, being compelled to content myself with advancing a few observations tending to elucidate positive laws of nature with which hitherto we have been but slightly acquainted.

First, then, utero-gestation, as to time, is entirely a question of age, and when this is understood, the term may with more certainty be fixed. Many cases have occurred in my own experience proving that, though utero-gestation varies in different individuals, *when the circumstances are analogous the time agrees.* Within the last twenty-five years I have attended eleven cases of labour completed as follows:—four before the 15th, and seven before the 16th year. The peculiar circumstances attending most of these cases admitted of nearly correct calculation as to time of impregnation, and they all apparently laboured at the full period of utero-gestation. In five, the gestation did not exceed 267 days. In two the account could not be depended upon, as the cohabitation was repeated more than once. the remaining four continued beyond the time calculated for such ages, but this apparent discrepancy can easily be reconciled by another conclusion arrived at on the subject, which I shall shortly enter upon, and more fully explain. I have also attended two cases of parturition occurring in the 52d year, in both of which the evidence as to time of impregnation may be considered correct, the utero-gestation extending in both cases to 290 days; and two cases of middle age—one at 25, which as nearly as could possibly be ascertained, extended to 274, and one at 35, to 278 days.

A most important series of experiments relating to this subject have been communicated to the world by the *Buffalo Medical Journal, U. S.*, which afford results similar to those I have here advanced.

Experiments on 621 cows—50 calved between 260 and 270 days.

| | | | | | |
|---|---|---|---|---|---|
| 556 | " | " | 270 | " | 280 " |
| 14 | " | " | 280 | " | 286 " |
| 1 | " | " | | | 290 " |

550 had mucous discharge for 24 hours, all were restless for 12 or 14 hours previous to labour, and all experienced regular pains. Those that calved before 270 days were *heifers* of their first calves. All that went over 228 days were *old cows* with large bellies; so that a cow *seven years old* will probably go 276 days. Experiments were tried of coition without the *heat*, but no impregnation followed.

The results of human gestation, also communicated by the same journal, are as follows—at the age of

| | |
|---|---|
| 17 | 270 days |
| 19 | 272 " |
| 30 | 276 " |
| 44 | 284 " |

If we combine the latter table with the result of my own observations just given, the following respective deductions (as to the term of utero-gestation in the human race) will be something nearer the truth, although very different to the conclusions previously arrived at by physiological writers generally.

| | | | |
|---|---|---|---|
| 15½ | years, | or near, | 267 days |
| 17 | " | " | 270 " |
| 19 | " | " | 272 " |
| 25 | " | " | 274 " |
| 30 | " | " | 276 " |
| 35 | " | " | 278 " |
| 44 | " | " | 284 " |
| 52 | " | " | 290 " |

This table shows plainly that the term of gestation is extended in proportion to the age of the parent; it is, however, but the result of a very limited number of cases; a wider experience, and a greater number of facts must be recorded before it is sufficiently confirmed as a general law, but it is hoped sufficient is here advanced to stimulate the members of our profession to further enquiry.

These results are very remarkable, and highly valuable in affording correct data on which to found a permanent system for calculating this important period. My own enquiries amongst intelligent cattle breeders fully confirm the statements of the American tables, and prove beyond a doubt that in domesticated animals, as for instance in the horse, cow, pig, and sheep, the time of gestation is of longer duration in the old, than in the younger animals; in fact, no difference of opinion exists upon the subject. This also applies to domestic fowls, the eggs of young hens being incubated twenty-four hours sooner than the eggs of old ones; a difference, according to the period of gestation, more remarkable than that occurring in man, or even in the quadrupeds already mentioned. From these circumstances it is evident that the duration of the term of gestation is definitive, certain as to time, unchangeable as all the laws of nature undoubtedly are, and regulated by that beautiful simplicity which characterizes all the physiology of conception, but hitherto unintelligible to the comprehension of man. If this is the case, (and the presumptive evidence is strong in its favour,) all that farrago of nonsense, abounding in our works of medical jurisprudence, is at once dissipated, and there remains a simple and definite law, which is regulated by age, and age alone. The very discrepancies and conflicting opinions of authors ought long ago to have led to this conclusion, but it is to be feared we are too much in the habit of seeking for solutions of disputed questions amidst the intricacies and difficulties of anatomical science, considering "omne ignotum pro magnifico," to be satisfied with explanations evident to all who will duly observe the undeviating and simple proofs afforded by nature herself.

Although it is evident that the consideration of this law leads to far more correct conclusions than any yet arrived at, difficulties still remain to be encountered; and except these are properly investigated, it is impossible that the desired correctness can be obtained. This leads us to consider the second law; which, in fact, ought to precede that already spoken of, simply because what we have denominated the first law is, really and truly, dependent on the second. To render this plainer and more easy of comprehension, I would lay down as an axiom that the term of utero-gestation is regulated by age, not of the mother only, but that it is dependent on the age of both paternal and maternal parent. I was first led to the consideration of this case by facts. Four of the eleven cases advanced in the early part of this paper, continued to a longer period than was justified by the calculation of the age of the mothers only; but what were the facts? *These four cases were impregnated by men considerably older than themselves* (two of the men being married, and fathers of families!) In other instances I observed this irregularity, but always attached to couples in which disparity of age existed. It then appeared to me to be only reasonable to consider the term of utero-gestation solely with reference to the ages of both parents. I therefore instituted a number of enquiries with the desire of ascertaining this point, and found that by striking an average of the two ages, and calculating accordingly, I was enabled to arrive at a more correct conclusion.

If for the purpose of rendering my meaning fully apparent, I illustrate a case in figures, the calculation will be as follows. Suppose a female of 20 cohabit with a man of 30, I expected to obtain the correct period of the term of utero-gestation, by calculating the average at the age of 25. Although this afforded a result approaching nearer to the truth than other systems, I speedily discovered that the average was not as correct as might it be; for presuming upon the generally acknowledged fact, that the female arrives at maturity somewhat earlier than the male, it is only reasonable that a corresponding allowance should be made in striking the averages,—thus, for instance, if a female of 20 cohabit with a man of 30, the term of utero-gestation will not be that of 25, but 24; and *vice versa*, for a female of 30 and a male of 20, will probably afford the result of 26 instead of 25. It will perhaps require a series of observations extending over many years, and a system of enquiry conducted by many individuals, to substantiate these positions, and probably some modifications may be necessary; still there are sufficient data already collected, to prove, that the term of utero-gestation is lengthened, or shortened, in proportion to the age of the parents. That this is dependent on the ages of both parents is confirmed by facts which I have frequently observed—viz., that a longer term of utero-gestation than was expected invariably occurred when a young female cohabited with an elderly male; and that a shorter period than was expected from the age of the female, elapsed, when the female, being of considerable age, had cohabited with a male much younger. Reasoning from analogy, these views are fully confirmed by the calculations formed on the observations of domestic animals previously quoted in this paper, and which are facts widely known and generally admitted, except by medical men in reference to the human being.—And why should it differ in the human female? Is it not infinitely more reasonable than the thirty-nine weeks, plus one day, of Dr. Blundell, applied to all ages and circumstances indiscriminately, and which every careful observer knows to be a fallacy? Every work of medical jurisprudence—every legal court record—nay, the private practice of every medical man disproves it.

There are many well-authenticated cases on record, which admit of no dispute, proving that the term of utero-gestation may be extended a few days from one extreme of life to another; but I boldly deny that gestation ever did, or could extend months, or even weeks beyond the usual term; that is when the fœtus and parent are normal in all their bearings. I also declare that two females of the same age, and similarly circumstanced, cohabiting with males of corresponding ages, will experience a similar extent of utero-gestation. These observations are the result of much diligent enquiry, afforded by an extensive practice of many years duration; and now that some few inaccuracies have been satisfactorily corrected, the more we endeavour to test their truth by further application, the more am I convinced of the correctness of their conclusions. I do not deny the necessity of continued enquiries and more general calculation, but I feel certain that the result will be to substantiate the truth and value of these observations, which are given in this condensed form, more with a view of stimulating to further inquiry than of building a truism on the few facts here given ; time and deep research alone can unravel the question, and place the legitimate value on the suggestions here put forward.

One word more respecting the different conclusions arrived at by various obstetric authorities, as to the term of utero-gestation. That they differ the following short summary will sufficiently prove. Dr. Ryan, 272 days.—Dr. Blundell, 274. A great number of authors fix the term at 280. Some again at 283. The laws of Prussia state 302. Code Napoleon, 300. Dr. Rigby cites three cases, one of rape, and two where the act of coition was only once in each, and the terms were at delivery 260, 264, and 276 days; and such differences will ever arise so long as menstrual periods are made the base of calculation ; no true period can be fixed upon, except in cases where sexual intercourse occurs but once (the time being known) and pregnancy resulting therefrom. It is evident the different conclusions arrived at by authors may be perfectly true *as individual cases*, but it is erroneous in the extreme to take *any given number of days as applicable to all cases at all ages.* For even where the sexual intercourse has been but once and pregnancy results, such cases vary some little, as asserted in the foregoing remarks, in consequence of the difference of age ; still that variation is less than under any other mode. It is the loose manner in which these calculations are made in connection with the last menstrual period that has led to the absurd doctrine entertained by some, that a female may go beyond the natural period assigned for utero-gestation ; thus for instance, if a female should happen to have the menstruation checked by some slight morbid interference (a simple cold) and should happen to become impregnated before the next period, for menstruation, such reasoners would have no other resource than to reckon from the last appearance, which (on account of the obstruction previously spoken of, by morbid interference,) would make it appear that the term of utero-gestation had extended to eleven months, when no such a phenomenon had occurred. In fact, I believe it impossible that an extension of utero-gestation (under circumstances perfectly normal in respect to mother and child) can occur for more than a few hours, or a few days, accounted for by the ages of the respective parties. I think it may be very positively asserted that all cases of rape and subsequent pregnancy, as well as authenticated cases of impregnation from one coition, the date of which being known, tend to disprove all the facts of protracted gestation except those referable to morbid interference. I now leave the subject to the consideration of the readers, at the same time assuring them that any facts in support of this new doctrine, or any decidedly negative proofs against it, will be thankfully received.—*British Record of Obstetric Medicine and Surgery.*

